# Circulating insulin-like growth factor-I and risk of 25 common conditions: outcome-wide analyses in the UK Biobank study

**DOI:** 10.1007/s10654-021-00811-y

**Published:** 2021-11-08

**Authors:** Keren Papier, Anika Knuppel, Aurora Perez-Cornago, Eleanor L. Watts, Tammy Y. N. Tong, Julie A. Schmidt, Naomi Allen, Timothy J. Key, Ruth C. Travis

**Affiliations:** 1 Cancer Epidemiology Unit, Nuffield Department of Population Health, University of Oxford, Richard Doll Building, Roosevelt Drive, Oxford, OX3 7LF UK; 2 Clinical Trial Service Unit and Epidemiological Studies Unit, Nuffield Department of Population Health, University of Oxford, Oxford, UK

**Keywords:** Insulin-like growth factor-I, Prospective cohort study, UK Biobank, Risk, Outcome-wide

## Abstract

While there is strong epidemiological evidence that circulating insulin-like growth factor-I (IGF-I) is associated with a higher risk of several cancers, little is known about its association with non-cancer outcomes. We investigated associations of circulating IGF-I with risk of 25 common conditions, other than cancer, in a large British cohort. Study participants were 318,749 middle-aged adults enrolled in the UK Biobank Study. Serum IGF-I concentration was measured in samples collected at baseline (2006–2010), and re-measured in 12,334 participants after an average of 4.3 years. We followed-up participants over an average of 11.5 years by linking to hospital admissions and mortality registries. Multivariable-adjusted Cox regressions estimated hazard ratios (HRs) and 95% confidence intervals (CIs) for associations between circulating IGF-I and 25 common conditions, using the repeated IGF-I measurements to correct for regression dilution bias. After correction for multiple testing (*P* < 0.002), IGF-I was positively associated with carpal tunnel syndrome (HR per 5 nmol/l higher concentration = 1.12, 95% CI 1.08–1.16), and inversely associated with varicose veins (0.90, 0.85–0.95), cataracts (0.97, 0.95–0.99), diabetes (0.92, 0.90–0.95), and iron deficiency anaemia (0.90, 0.86–0.93). The associations for cataracts and diabetes attenuated when restricted to cases diagnosed after five or more years of follow-up, suggesting that these associations were likely affected by reverse causality. Higher IGF-I concentration might be associated with the risk for several conditions, but genetic studies are needed to clarify which associations may be causal.

**Supplementary Information:**

The online version contains supplementary material available at 10.1007/s10654-021-00811-y.

## Introduction

Insulin-like growth factor-I (IGF-I) is a polypeptide hormone that is primarily synthesised in the liver following growth hormone stimulation [1], and promotes tissue growth and development in multiple organ systems by acting as a primary mediator of the effects of growth hormone [2]. Clinically high circulating IGF-I concentration, as in adults with acromegaly, is associated with a higher risk of several diseases [3], particularly higher risks of cardiovascular disease, metabolic disorders (e.g. insulin resistance), biliary diseases (e.g. gallbladder disease), gastrointestinal diseases (e.g. colon polyps), arthropathy, musculoskeletal disorders (e.g. carpal tunnel syndrome), genitourinary diseases (e.g. kidney stones, enlarged prostate [4]), respiratory disease, sleep apnoea, and some cancers [5, 6].

Higher IGF-I concentrations in adults without acromegaly have been shown to also be associated with increased risks of several cancers [7], but corresponding evidence for non-cancer outcomes is inconsistent and/or limited to cross-sectional design. Although some of the available prospective observational and genetic evidence suggests that higher IGF-I levels might be positively associated with type 2 diabetes [8, 9], ischaemic heart disease (IHD) [9–11], hip and knee osteoarthritis [12], enlarged prostate [13], and colon adenomas [14, 15], some studies also reported null [16–24], and inverse [25] associations for these outcomes. These equivocal findings and the lack of available prospective evidence for many non-cancer outcomes likely relates at least in part to outcome-selection bias, small sample sizes, and/or the relatively short follow-up of previous studies. To address this, we used an outcome-wide approach to examine the prospective association of circulating IGF-I with 25 common, non-cancerous diseases and conditions, in a large British study of men and women with over 10 years of follow-up.

## Methods

### Study population

The UK Biobank is a prospective cohort study of middle-aged men and women (aged 40–69 years at recruitment) from the general population across the UK [26]. Approximately 9.2 million individuals who were registered with the National Health Service (NHS) and lived within about 40 km of one of 22 UK Biobank assessment centres were invited to participate, of whom 503,317 (5.5%) joined the cohort between 2006 and 2010 [27].

### Laboratory assessment

All participants provided a non-fasting blood sample at recruitment and approximately 20,000 participants (21% of those re-invited) agreed to provide an additional blood sample as part of a repeat assessment between 2012 and 2013 (https://biobank.ctsu.ox.ac.uk/~bbdatan/Repeat_assessment_doc_v1.0.pdf). IGF-I concentration was measured in serum samples which had been stored at − 80 °C, using chemiluminescent immunoassays (DiaSorin Liaison XL, analytical range 1.3–195 nmol/l). [28] The average within-laboratory coefficients of variation (ratio of the standard deviation to the mean for quality control samples) ranged from 5.3 to 6.2%. Full details of the assay methods and quality assurance protocols are available online (https://biobank.ndph.ox.ac.uk/showcase/showcase/docs/serum_biochemistry.pdf).

### Assessment of other characteristics

Participants provided information on their personal, physical, sociodemographic and other lifestyle characteristics at the baseline assessment visit via a self-completed touchscreen questionnaire and a computer assisted personal interview. Participants also underwent physical measurements (e.g. weight and height) during the recruitment visit.

### Assessment of health outcomes

Participants’ health was followed up via record linkage to routine health records, including national death and cancer registers and in-patient hospital admissions. Outcomes of interest were the 25 most common, well-defined primary causes of non-cancer related hospital admission in this cohort based on the primary International Classification of Diseases (ICD) 10 diagnosis codes recorded during admission. We excluded some common reasons for hospital admission in this cohort (e.g. nausea or heartburn) because they were not well-defined and/or were likely to be associated with a diverse range of underlying conditions. We included diabetes even though it was not among the 25 most common primary diagnoses associated with admission, as it is a common secondary (i.e. co-incident or underlying) reason for admission (See Supplementary Methods M1 and Supplementary Table S1 for information on censor dates, diagnosis, and procedure codes.) The outcomes selected for the present study were guided by previous work with this cohort [29] due to the lack of knowledge regarding associations between IGF-I and many non-cancer outcomes and the exploratory nature of this work.

### Exclusions

Of the 503,317 participants recruited, 829 were excluded because they had withdrawn from the study, 4066 were excluded because they had no biomarker data at all, and 31,397 were excluded because they had missing data for IGF-I concentration at baseline. We additionally excluded participants whose genetic sex differed from their reported gender (*n* = 355), those with missing data for weight (*n* = 1553) or height (*n* = 307), prevalent cancer (except non-melanoma skin cancer, ICD-10 C44) prior to recruitment (*n* = 24,626), those who did not self-report good or excellent health (*n* = 110,830), those with prevalent diabetes or unknown diabetes status (*n* = 10,425), and those with less than one year of follow-up (*n* = 180) to reduce the risk of reverse causality, resulting in study sample of 318,749 participants. (Supplementary Fig. S1).

### Statistical analysis

We used Cox proportional hazards regression models, with age as the underlying time variable, to estimate hazard ratios (HRs) and 95% confidence intervals (CIs) for associations between IGF-I concentration and each condition of interest, with Bonferroni correction to allow for multiple testing (*P* < 0.002 for 25 tests). IGF-I concentrations were modelled both continuously (per 5 nmol/l of IGF-I concentration, equivalent to ~ 1 SD in the cohort) and categorically (sex-specific fifths). Each endpoint was assessed using a separate analysis with participants contributing person-years at-risk for each condition of interest for that analysis. We calculated person-years of follow-up using participants’ age at recruitment and their age at hospital admission, death, or loss or end of follow up (November 30th, 2020 for England, October 31st, 2020 for Scotland, and February 28th, 2018 for Wales).

The use of a single measure can lead to under-estimation of risks due to within-person variability and laboratory measurement error (“regression dilution bias”) [30]. Therefore, we used the repeated IGF-I measures collected from 12,334 participants (who met our inclusion criteria) on average 4.3 years (SD 0.9 years) after recruitment to correct HRs for trend using the McMahon-Peto method [31]; the log HRs and standard errors were divided by the sex-specific regression dilution ratios from the subsample, which were obtained by dividing the difference in the mean IGF-I concentrations between the 5th and 1st fifths in the repeat measure by the equivalent mean difference in the baseline measure.

All analyses were stratified by age group (< 45, 45–49, 50–54, 55–59, 60–64, ≥ 65 years), sex and geographical region (London, North–West, North–East, Yorkshire and Humber, West Midlands, East Midlands, South–East, South–West, Wales, and Scotland) (Model 0). In Model 1, we additionally adjusted for ethnicity (White, non-White, unknown), socio-economic deprivation (Townsend index quintiles, unknown), qualification (college or university degree/vocational qualification, national examination at ages 17–18 years, national examination at age 16 years, other or unknown), smoking (never, former, current < 15 cigarettes/day, current > 15 cigarettes/ day, unknown), physical activity (< 10, 10–19, 20–39, 40–59, ≥ 60 MET hours per week, unknown), alcohol intake (< 1.0, 1.0–4.9, 5.0–9.9, 10.0–14.9, 15.0–19.9, 20.0–24.9, and ≥ 25.0 g/day, non-drinker, and unknown), and height (continuous). For women, Model 1 was additionally adjusted for menopausal status (pre-, postmenopausal, unknown), hormone-replacement therapy (HRT) use (never, past, current, unknown), oral contraceptive pill (OCP) use (never, past, current, unknown), and parity (nulliparous, 1–2, 3 or more, unknown). For Model 2, we further adjusted for body mass index (BMI) (< 20.0, 20.0–22.49, 22.5–24.99, 25.0–27.49, 27.5–29.99, 30.0–32.49, 32.5–34.99, > 35.0 kg/m^2^). An ‘unknown’ category was created for each covariate with missing data (proportion of missings ranged from < 1% for alcohol to 20% for physical activity).

### Sensitivity analyses

We assessed the potential for residual confounding by other relevant biomarkers previously found to be associated with IGF-I and some of the outcomes [32, 33] by additionally adjusting our main model (Model 2) for serum concentrations of C-reactive protein, glycated haemoglobin, and sex hormone–binding globulin (fifths, unknown for each) (Model 3). We also assessed heterogeneity by follow-up time at diagnosis (less than five years and five years or over) using stratified Cox regression models, comparing HRs and standard errors in the two subgroups using a *χ*^2^ test for heterogeneity (*P* < 0.05).

All analyses were conducted using STATA version 16.1 (Stata Corp LP, College Station, TX, USA). All *P* values were two-sided.

## Results

### Baseline characteristics

Table 1 presents the cohort characteristics of study participants by mean baseline levels of circulating IGF-I. We observed higher IGF-I concentrations in men, younger adults, those who self-identified to be of non-White ethnicity, those in the top fifth of height, adults with a BMI in the middle range (between 22.5 and 27.5 kg/m^2^) compared to those with a lower and higher BMI, adults who were affluent and had a higher level of attained education, non-smokers, moderate alcohol consumers, adults with low levels of physical activity, and women who had never used hormone replacement therapy, were using the oral contraceptive pill, did not have children, and were premenopausal. At baseline, the mean circulating IGF-I concentration was 21.7 nmol/l (SD 5.5). The correlation coefficient between IGF-I measured at baseline and at follow-up was 0.77.

**Table 1:** Baseline characteristics by serum IGF-I levels in UK Biobank participants (*n* = 318,749)

Characteristics	*n*	Circulating IGF-I concentration (nmol/l), mean (SD)
IGF-I concentration at baseline	318,749	21.7 (5.5)
Repeat IGF-I concentration	12,334	21.2 (5.4)
*Sex*		
Women	178,317	21.3 (5.6)
Men	140,432	22.2 (5.3)
*Age at baseline (years)*		
39–44	34,823	24.8 (5.5)
45–49	43,854	23.3 (5.4)
50–54	49,466	22.2 (5.4)
55–59	57,693	21.4 (5.3)
60–64	76,039	20.7 (5.2)
65–73	56,874	20.0 (5.2)
*Ethnicity*		
White	304,496	21.7 (5.5)
Non-white	13,295	22.1 (5.9)
Unknown	958	21.5 (5.4)
*Standing height (quintiles)*		
Lowest (Q1)	64,981	20.7 (5.5)
Highest (Q5)	59,493	22.8 (5.4)
*Body mass index (kg/m*^*2*^*)*		
< 20.0	8298	21.0 (5.5)
20.0–22.49	37,777	21.9 (5.5)
22.5–24.99	75,549	22.1 (5.5)
25.0–27.49	82,540	22.1 (5.4)
27.5–29.99	57,609	21.8 (5.5)
30.0–32.49	30,598	21.2 (5.5)
32.5–34.99	14,279	20.4 (5.6)
> 35.0	12,099	19.2 (5.5)
*Socio-economic status (Townsend deprivation index)*		
Most affluent (Q1)	70,381	21.9 (5.5)
Most deprived (Q5)	52,155	21.5 (5.7)
Unknown	355	22.0 (5.2)
*Qualification*		
National examination at age 16 years	52,472	21.6 (5.5)
National examination at ages 17/18 years	18,116	22.0 (5.6)
College or university degree/vocational qualification	202,072	22.0 (5.5)
Other	43,476	20.4 (5.3)
Unknown	2613	21.0 (5.7)
*Smoking*		
Non-smoker	184,160	22.0 (5.6)
Former smoker	106,850	21.3 (5.4)
Current smoker, < 15 cigarettes/day	8161	21.5 (5.6)
Current smoker, ≥ 15 cigarettes /day	18,539	21.5 (5.5)
Unknown	1039	20.7 (5.3)
*Alcohol intake (grams/day)*		
Non-drinker	19,520	21.0 (5.8)
< 1.0	31,501	21.4 (5.9)
1–4.9	55,042	21.9 (5.7)
5–9.9	49,019	22.0 (5.5)
10–14.9	49,673	22.0 (5.5)
15–19.9	25,939	22.0 (5.3)
20–24.9	26,346	22.0 (5.3)
≥ 25	61,538	21.2 (5.2)
Unknown	171	20.8 (5.3)
*Physical activity (metabolic equivalent hours/wk)*		
< 10	53,658	21.8 (5.6)
10–19	52,821	22.2 (5.5)
20–39	67,948	22.0 (5.5)
40–59	34,025	21.8 (5.5)
≥ 60	47,499	21.5 (5.4)
Unknown	62,798	21.2 (5.5)
*Hormone replacement therapy use (in women)*		
Never	113,838	22.0 (5.7)
Past	53,507	20.3 (5.3)
Current	10,543	18.9 (5.5)
Unknown	429	21.8 (6.1)
*Oral contraceptive pill use (in women)*		
Never	31,315	20.5 (5.5)
Past	142,963	21.4 (5.6)
Current	3667	25.2 (6.3)
Unknown	372	20.9 (6.2)
*Parity (in women)*		
Nulliparous	33,732	21.9 (5.8)
1.0–2.0	103,228	21.4 (5.6)
≥ 3	41,247	20.7 (5.5)
Unknown	110	20.5 (6.0)
*Menopausal status (in women)*		
Pre-	44,525	23.8 (5.6)
Post-	124,691	20.4 (5.4)
Unknown	9101	22.2 (5.8)

*IGF-I* insulin-like growth factor-I, *Q1* lowest quintile, *Q5* highest quintile, *SD* standard deviation

### Risk analyses

**Fig. 1:**
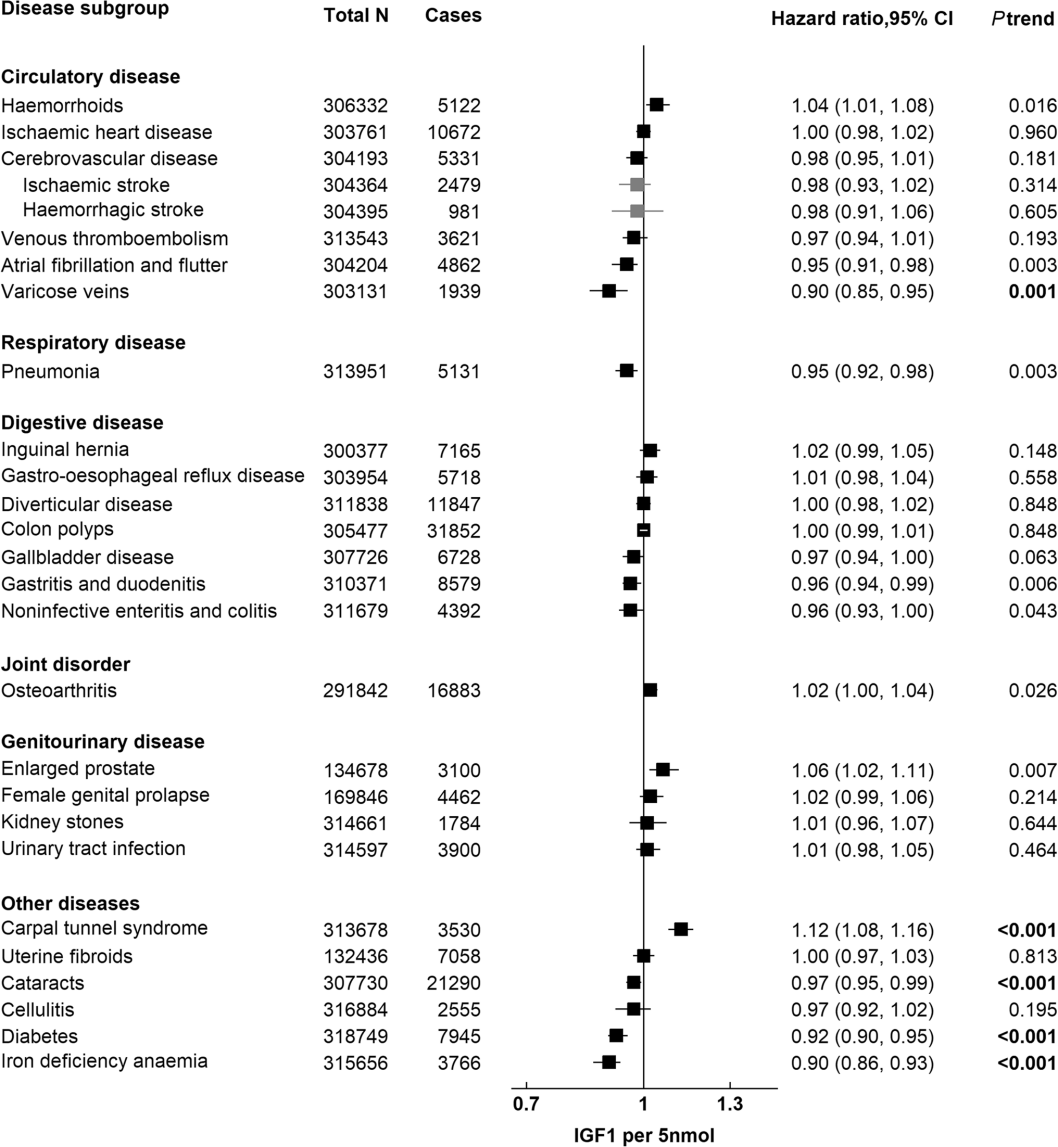
Relative risk of 25 common conditions per 5 nmol/l higher IGF-I concentration in UK Biobank, corrected for regression dilution bias**.** Stratified for age group (< 45, 45–49, 50–54, 55–59, 60–64, and ≥ 65 years), sex and region and adjusted for age (underlying time variable), ethnicity (White, non-white, unknown), deprivation (Townsend index quintiles, unknown), qualification (College or university degree/vocational qualification, National examination at ages 17–18,National examination at age 16, other or unknown), smoking (never, former, current < 15 cigarettes/day, current > 15 cigarettes/ day, unknown), physical activity (< 10, 10– < 20, 20–40, 40- < 60, >  = 60 MET hours per week, unknown), alcohol intake (< 1.0, 1.0–4.9, 5.0–9.9, 10.0–14.9, 15.0–19.9, 20.0–24.9, and ≥ 25.0 g/day, non-drinker, and unknown), height (continuous), and in women: menopausal status (pre-, postmenopausal, unknown), hormone-replacement therapy (never, past, current, unknown), oral contraceptive pill intake (never, past, current, unknown), parity (nulliparous, 1–2, 3 or more, unknown), and body mass index (< 20.0, 20.0–22.49, 22.5–24.99, 25.0–27.49, 27.5–29.99, 30.0–32.49, 32.5–34.99, > 35.0). CI confidence intervals, IGF-I insulin-like growth factor-I. *P* trend in bold: *P* value statistically significant after Bonferroni correction (*P* < 0.002)

Figure 1 presents the HRs and 95% CIs for 25 common conditions in relation to a per 5 nmol/l higher serum IGF-I concentration, ordered by disease subgroup (circulatory, respiratory, digestive, joint disorder, genitourinary and other diseases) and effect size; estimated using multivariable-adjusted Cox regression models (Model 2), corrected for regression dilution bias. After correction for multiple testing (*P* < 0.002), there was a positive association between IGF-I concentration and carpal tunnel syndrome (HR per 5 nmol/l higher concentration = 1.12, 95% CI, 1.08–1.16), and inverse associations with varicose veins (0.90, 0.85–0.95), cataracts (0.97, 0.95–0.99), diabetes (0.92, 0.90–0.95), and iron deficiency anaemia (0.90, 0.86–0.93). Similar associations were observed by sex-specific fifths of IGF-I concentration for these outcomes: carpal tunnel syndrome (HR in the top versus the lowest fifth = 1.34, 95% CI, 1.20–1.50), varicose veins (0.75, 0.64–0.87), cataracts (0.91, 0.87–0.95), diabetes (0.85, 0.79–0.91), and iron deficiency anaemia (0.77, 0.69–0.85) (Supplementary Table S2). Higher IGF-I concentration was also associated with lower risks of atrial fibrillation and flutter, pneumonia, gastritis and duodenitis, and noninfective enteritis and colitis, and higher risks of haemorrhoids, osteoarthritis, and enlarged prostate at the *P* < 0.05 level.

### Sensitivity analyses

We observed similar results to those from the main analysis in sensitivity analyses adjusting for serum concentrations of C-reactive protein, glycated haemoglobin, and sex hormone–binding globulin (Supplementary Table S2, Model 3).

**Fig. 2:**
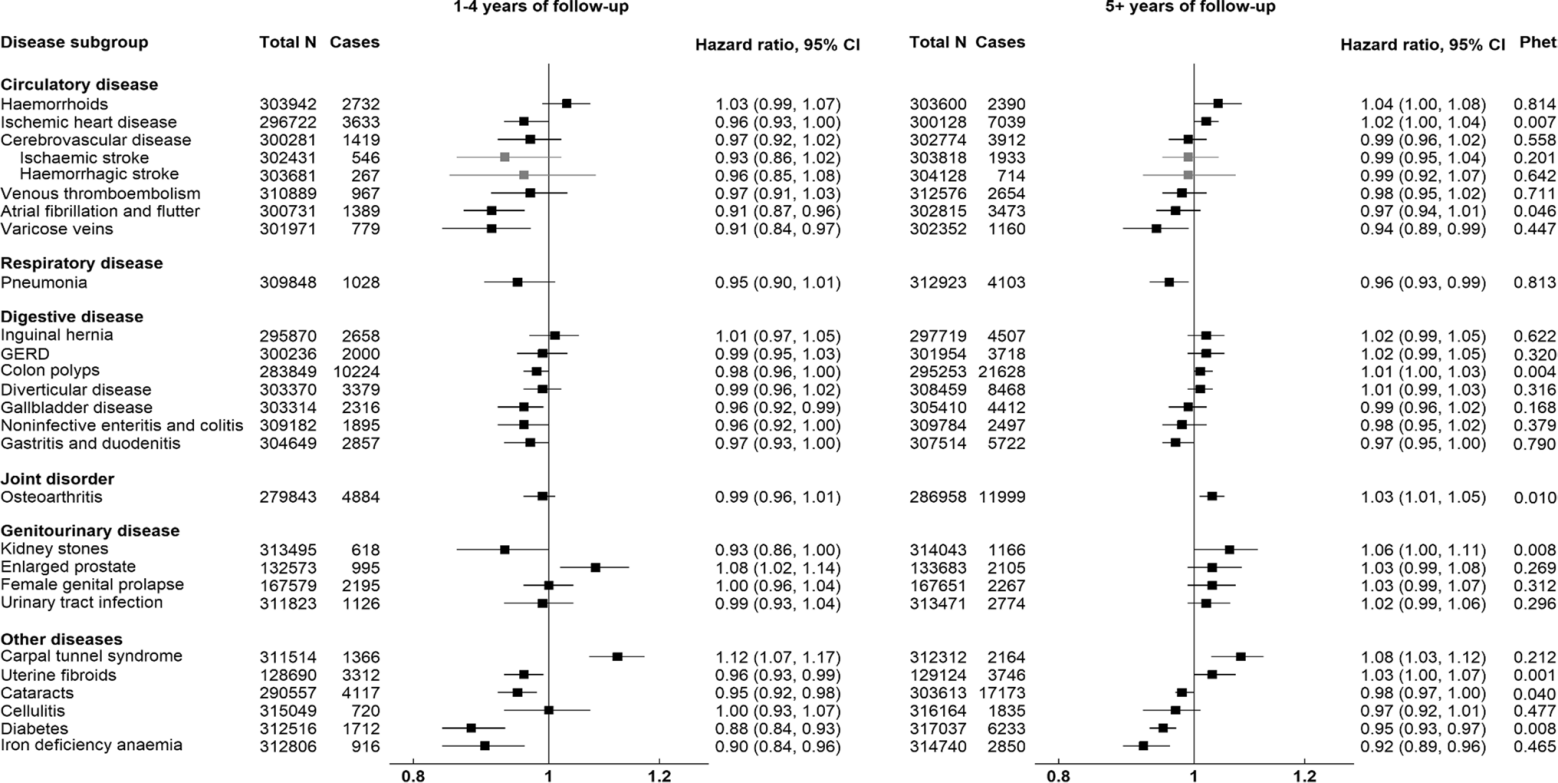
Relative risk of 25 common conditions per 5 nmol/l higher IGF-I concentration by follow-up time at diagnosis. Stratified for age group (< 45, 45–49, 50–54, 55–59, 60–64, and ≥ 65 years), sex and region and adjusted for age (underlying time variable), ethnicity (White, non-white, unknown), deprivation (Townsend index quintiles, unknown), qualification (College or university degree/vocational qualification, National examination at ages 17–18,National examination at age 16, other or unknown), smoking (never, former, current < 15 cigarettes/day, current > 15 cigarettes/ day, unknown), physical activity (< 10, 10- < 20, 20–40, 40- < 60, >  = 60 MET hours per week, unknown), alcohol intake (< 1.0, 1.0–4.9, 5.0–9.9, 10.0–14.9, 15.0–19.9, 20.0–24.9, and ≥ 25.0 g/day, non-drinker, and unknown), height (continuous), and in women: menopausal status (pre-, postmenopausal, unknown), hormone-replacement therapy (never, past, current, unknown), oral contraceptive pill intake (never, past, current, unknown), parity (nulliparous, 1–2, 3 or more, unknown), and body mass index (< 20.0, 20.0–22.49, 22.5–24.99, 25.0–27.49, 27.5–29.99, 30.0–32.49, 32.5–34.99, > 35.0). GERD Gastro-oesophageal reflux disease, CI confidence intervals, IGF-I insulin-like growth factor-I

Figure 2 shows associations between risk of 25 common conditions and higher IGF-I concentration (per 5 nmol/l) stratified by follow-up time at diagnosis (less than five years and five years or over). There was evidence of heterogeneity by follow-up time for associations with cataracts (Phet = 0.040) and diabetes (Phet = 0.008), with associations closer to null in participants diagnosed after five or more years of follow-up. We also observed positive associations for IHD, haemorrhoids, colon polyps, osteoarthritis, kidney stones, and uterine fibroids, and an inverse association for pneumonia in participants diagnosed after five or more years of follow-up (Phet < 0.05).

## Discussion

In this large prospective and outcome-wide investigation of associations between circulating IGF-I and a range of non-cancer outcomes, we found that, after accounting for multiple testing and regression dilution bias, higher IGF-I concentration was positively associated with risk of carpal tunnel syndrome and inversely associated with incident varicose veins, cataracts, diabetes, and iron deficiency anaemia. The associations for carpal tunnel syndrome, varicose veins, and iron deficiency anaemia did not vary by follow-up time at diagnosis. For cataracts and diabetes though, the associations were closer to null in those diagnosed after five or more years of follow-up, suggesting that these associations might have been affected by reverse causality (whereby IGF-I levels change as a result of early pathophysiological processes).

To our knowledge, this is the first study of IGF-I and risk of carpal tunnel syndrome in a general, population-based cohort. In line with our results, one case–control study found that 34 adults with acromegaly had larger peripheral nerves, and that biochemical control of IGF-I concentrations over a one-year follow-up resulted in reduced nerve size [34]. The positive association between circulating IGF-I and carpal tunnel syndrome could be plausible due to IGF-I’s involvement in nerve growth and formation [35]; in adults with acromegaly, carpal tunnel syndrome has been attributed to median nerve enlargement, which was correlated with circulating IGF-I [36, 37].

We found a small inverse association between IGF-I and risk of varicose veins. This is a novel epidemiological finding, although an *in-vitro* study found that IGF-I was associated with the proliferation of smooth muscle cells of saphenous veins [38]. IGF-I’s role in promoting the growth and survival of smooth muscle cells would suggest that the inverse association we observed is plausible.

We also found an inverse association between IGF-I concentration and iron deficiency anaemia. We are not aware of any published prospective evidence, but previous cross-sectional studies have found that low IGF-I concentrations were associated with lower haemoglobin concentration and higher prevalence of anaemia in middle-aged and elderly adults [39–42]. IGF-I might play an important role in erythropoiesis [43], suggesting that the observed inverse association is plausible. However, iron deficiency anaemia might have a longer lag time from disease onset or initial diagnosis to hospital admission [44], therefore reverse causality cannot be ruled out and longer follow-up is needed.

We found a novel inverse association between IGF-I and risk of cataracts. However, in analyses stratified by follow-up time at diagnosis this appeared to be due to reverse causality, possibly related to a shared pathophysiology with insulin resistance. In support of our findings, in adults with acromegaly, visual disturbances appear to relate to the effects of space-occupying lesions of pituitary adenomas rather than to circulating IGF-I levels [45]. Also, some evidence from in-vitro rat models has shown that IGF-I might decrease the amount of α-crystallin (a lens protein) made in the lens fibre cells [46], therefore this might be expected to lead to a positive association of IGF-I with risk of cataracts since higher levels of α-crystallin have been associated with a lower risk of cataract formation [46].

Recent prospective and genetic evidence has suggested that there could be a positive association between circulating IGF-I concentration and type 2 diabetes risk [8, 9, 16], possibly due to its involvement in glucose homeostasis [47]. This is in contrast to the inverse association we observed for incident diabetes (which is likely to be mostly type 2 diabetes due to the older age structure of this cohort). However, our findings may be due to reverse causality. Diabetes may go undiagnosed for years [48], therefore a long follow-up period is needed to avoid picking-up prevalent cases or pre-clinical disease.

We also found evidence for positive associations between IGF-I and IHD, haemorrhoids, colon polyps, osteoarthritis, kidney stones, and uterine fibroids, and an inverse association with pneumonia in participants diagnosed after five or more years of follow-up. The findings for IHD [9], osteoarthritis [12], colon polyps [14, 15], and uterine fibroids[49] are in line with some of the available prospective or genetic evidence from population-based studies, and the results for kidney stones are supported by studies in adults with acromegaly [5]. It is possible that some of these associations were masked by reverse causality in the first five years of follow-up, and follow-up with larger numbers is needed to clarify whether IGF-I might associate with these outcomes.

This is the first study to adopt an outcome-wide approach to the investigation of IGF-I and risk of 25 common conditions (other than cancer); this comprehensive approach allowed us to assess and compare the effect sizes of multiple outcomes within the UK Biobank and reduce outcome-selection bias. Additional strengths of our study include its population-based design, the use of national record linkage to ascertain information on disease incidence, which eliminates misclassification and reduces attrition bias at follow-up, and its large size; this is the largest prospective study of IGF-I and most of these 25 common conditions.

Nevertheless, our study is not without its limitations. Some measurement error could have occurred when measuring IGF-I at baseline, but we reduced the potential impact of regression dilution bias by correcting baseline measures in all participants with a repeat IGF-I measure from a subsample of participants. Furthermore, we cannot rule-out that multiple testing could have led to some chance findings, though we addressed this using Bonferroni correction. However, this is a strict approach and it is possible that some of the associations with a *P* < 0.05 do not reflect a chance finding. Additionally, because we used an outcome-wide approach, it is possible that we did not fully adjust for confounders that might have affected some of the individual conditions. However, many of these associations are exploratory and therefore not all of the confounders are known. Moreover, some conditions might go undiagnosed for some time and only require hospital care at later stages, and therefore might reflect prevalent or preclinical disease and/or more severe disease (such as in the case of diabetes). Also, we did not exclude participants with prevalent acromegaly at baseline; acromegaly is associated with higher IGF-I and several diseases [3] but it is very rare (with the prevalence ranging from 85 to 133 cases per million in Europe [50], and estimated to be < 1% in the UK Biobank) and therefore unlikely to have had a large impact on the estimates presented. Furthermore, IGF-I–related proteins such as IGF-II and IGF-binding proteins (IGFBP), which play a role in the regulation of IGF-I bioavailability and signalling [51], were not measured in this study. Therefore, the observed associations could partially reflect other aspects of the IGF signalling pathway. Finally, the UK Biobank is predominantly made up of white Europeans, so the generalisability of our findings might be limited.

## Conclusions

The findings from this large population-based study suggest that circulating IGF-I may be associated with several common conditions (other than cancer), including positively with risk of carpal tunnel syndrome and inversely with risks of varicose veins and iron deficiency anaemia. Additional studies, including genetic analyses, are needed to assess whether these findings reflect casual relationships.

## Supplementary Information

Below is the link to the electronic supplementary material.

Supplementary file1 (DOCX 78 kb)

## Data Availability

The data, codebook and analytic code described in the manuscript will be made available for bona fide researchers who apply to use the UK Biobank data set by registering and applying at http://www.ukbiobank.ac.uk/register-apply.

## References

[1] Melmed S (2009). Acromegaly pathogenesis and treatment. J Clin Investig.

[2] Pollak MN, Schernhammer ES, Hankinson SE (2004). Insulin-like growth factors and neoplasia. Nat Rev Cancer.

[3] Sherlock M, Ayuk J, Tomlinson JW, Toogood AA, Aragon-Alonso A, Sheppard MC (2010). Mortality in patients with pituitary disease. Endocr Rev.

[4] Watts EL, Goldacre R, Key TJ, Allen NE, Travis RC, Perez-Cornago A (2020). Hormone-related diseases and prostate cancer: an English national record linkage study. Int J Cancer.

[5] Parolin M, Dassie F, Vettor R, Maffei P (2020). Acromegaly and ultrasound: how, when and why?. J Endocrinol Invest.

[6] Katznelson L, Laws ER, Melmed S, Molitch ME, Murad MH, Utz A (2014). Acromegaly: an endocrine society clinical practice guideline. J Clin Endocrinol Metab.

[7] Knuppel A, Fensom GK, Watts EL, Gunter MJ, Murphy N, Papier K (2020). Circulating insulin-like growth factor-I concentrations and risk of 30 cancers: prospective analyses in UK Biobank. Can Res.

[8] Rajpathak SN, He M, Sun Q, Kaplan RC, Muzumdar R, Rohan TE (2012). Insulin-like growth factor axis and risk of type 2 diabetes in women. Diabetes.

[9] Larsson SC, Michaëlsson K, Burgess S (2020). IGF-1 and cardiometabolic diseases: a Mendelian randomisation study. Diabetologia.

[10] Carlzon D, Svensson J, Petzold M, Karlsson MK, Ljunggren Ö, Tivesten Å (2014). Both low and high serum IGF-1 levels associate with increased risk of cardiovascular events in elderly men. J Clin Endocrinol Metab.

[11] Page JH, Ma J, Pollak M, Manson JE, Hankinson SE (2008). Plasma insulinlike growth factor 1 and binding-protein 3 and risk of myocardial infarction in women: a prospective study. Clin Chem.

[12] Hartley A, Sanderson E, Paternoster L, Teumer A, Kaplan RC, Tobias JH (2020). Mendelian randomization provides evidence for a causal effect of higher serum IGF-1 concentration on risk of hip and knee osteoarthritis. Rheumatology (Oxford).

[13] Chokkalingam AP, Gao YT, Deng J, Stanczyk FZ, Sesterhenn IA, Mostofi FK (2002). Insulin-like growth factors and risk of benign prostatic hyperplasia. Prostate.

[14] Soubry A, Il'yasova D, Sedjo R, Wang F, Byers T, Rosen C (2012). Increase in circulating levels of IGF-1 and IGF-1/IGFBP-3 molar ratio over a decade is associated with colorectal adenomatous polyps. Int J Cancer.

[15] Giovannucci E, Pollak MN, Platz EA, Willett WC, Stampfer MJ, Majeed N (2000). A prospective study of plasma insulin-like growth factor-1 and binding protein-3 and risk of colorectal neoplasia in women. Cancer Epidemiol Biomarkers Prev.

[16] Zanetti D, Gustafsson S, Assimes TL, Ingelsson E (2020). Comprehensive investigation of circulating biomarkers and their causal role in atherosclerosis-related risk factors and clinical events. Circ Genom Precis Med.

[17] Drogan D, Schulze MB, Boeing H, Pischon T (2016). Insulin-like growth factor 1 and insulin-like growth factor-binding protein 3 in relation to the risk of type 2 diabetes mellitus: results from the EPIC-Potsdam study. Am J Epidemiol.

[18] Similä ME, Kontto JP, Virtamo J, Hätönen KA, Valsta LM, Sundvall J (2019). Insulin-like growth factor I, binding proteins -1 and -3, risk of type 2 diabetes and macronutrient intakes in men. Br J Nutr.

[19] Ricketts SL, Rensing KL, Holly JM, Chen L, Young EH, Luben R (2011). Prospective study of insulin-like growth factor-I, insulin-like growth factor-binding protein 3, genetic variants in the IGF1 and IGFBP3 genes and risk of coronary artery disease. Int J Mol Epidemiol Genet.

[20] Harman SM, Metter EJ, Blackman MR, Landis PK, Carter HB (2000). Serum levels of insulin-like growth factor I (IGF-I), IGF-II, IGF-binding protein-3, and prostate-specific antigen as predictors of clinical prostate cancer. J Clin Endocrinol Metab.

[21] Mantzoros CS, Tzonou A, Signorello LB, Stampfer M, Trichopoulos D, Adami HO (1997). Insulin-like growth factor 1 in relation to prostate cancer and benign prostatic hyperplasia. Br J Cancer.

[22] Stattin P, Kaaks R, Riboli E, Ferrari P, Dechaud H, Hallmans G (2001). Circulating insulin-like growth factor-I and benign prostatic hyperplasia—a prospective study. Scand J Urol Nephrol.

[23] Zhai G, Rivadeneira F, Houwing-Duistermaat JJ, Meulenbelt I, Bijkerk C, Hofman A (2004). Insulin-like growth factor I gene promoter polymorphism, collagen type II alpha1 (COL2A1) gene, and the prevalence of radiographic osteoarthritis: the Rotterdam Study. Ann Rheum Dis.

[24] Fraenkel L, Zhang Y, Trippel SB, McAlindon TE, LaValley MP, Assif A (1998). Longitudinal analysis of the relationship between serum insulin-like growth factor-I and radiographic knee osteoarthritis. Osteoarthritis Cartilage.

[25] Sandhu MS, Heald AH, Gibson JM, Cruickshank JK, Dunger DB, Wareham NJ (2002). Circulating concentrations of insulin-like growth factor-I and development of glucose intolerance: a prospective observational study. Lancet.

[26] Collins R (2012). What makes UK Biobank special?. Lancet.

[27] Fry A, Littlejohns TJ, Sudlow C, Doherty N, Adamska L, Sprosen T (2017). Comparison of sociodemographic and health-related characteristics of UK biobank participants with those of the general population. Am J Epidemiol.

[28] Elliott P, Peakman TC (2008). The UK Biobank sample handling and storage protocol for the collection, processing and archiving of human blood and urine. Int J Epidemiol.

[29] Papier K, Fensom GK, Knuppel A, Appleby PN, Tong TYN, Schmidt JA (2021). Meat consumption and risk of 25 common conditions: outcome-wide analyses in 475,000 men and women in the UK Biobank study. BMC Med.

[30] Clarke R, Shipley M, Lewington S, Youngman L, Collins R, Marmot M (1999). Underestimation of risk associations due to regression dilution in long-term follow-up of prospective studies. Am J Epidemiol.

[31] MacMahon S, Peto R, Cutler J, Collins R, Sorlie P, Neaton J (1990). Blood pressure, stroke, and coronary heart disease. Part 1, prolonged differences in blood pressure: prospective observational studies corrected for the regression dilution bias. Lancet.

[32] Brunner EJ, Kivimäki M, Witte DR, Lawlor DA, Davey Smith G, Cooper JA (2008). Inflammation, insulin resistance, and diabetes–Mendelian randomization using CRP haplotypes points upstream. PLoS Med.

[33] Kalme T, Seppälä M, Qiao Q, Koistinen R, Nissinen A, Harrela M (2005). Sex hormone-binding globulin and insulin-like growth factor-binding protein-1 as indicators of metabolic syndrome, cardiovascular risk, and mortality in elderly men. J Clin Endocrinol Metab.

[34] Resmini E, Tagliafico A, Nizzo R, Bianchi F, Minuto F, Derchi L (2009). Ultrasound of peripheral nerves in acromegaly: changes at 1-year follow-up. Clin Endocrinol (Oxf).

[35] Rabinovsky ED (2004). The multifunctional role of IGF-1 in peripheral nerve regeneration. Neurol Res.

[36] Sasagawa Y, Tachibana O, Doai M, Tonami H, Iizuka H (2015). Median nerve conduction studies and wrist magnetic resonance imaging in acromegalic patients with carpal tunnel syndrome. Pituitary.

[37] Tagliafico A, Resmini E, Nizzo R, Bianchi F, Minuto F, Ferone D (2008). Ultrasound measurement of median and ulnar nerve cross-sectional area in acromegaly. J Clin Endocrinol Metab.

[38] Jia G, Mitra AK, Gangahar DM, Agrawal DK (2010). Insulin-like growth factor-1 induces phosphorylation of PI3K-Akt/PKB to potentiate proliferation of smooth muscle cells in human saphenous vein. Exp Mol Pathol.

[39] Succurro E, Arturi F, Caruso V, Rudi S, Sciacqua A, Andreozzi F (2011). Low insulin-like growth factor-1 levels are associated with anaemia in adult non-diabetic subjects. Thromb Haemost.

[40] Nilsson-Ehle H, Bengtsson BA, Lindstedt G, Mellström D (2005). Insulin-like growth factor-1 is a predictor of blood haemoglobin concentration in 70-yr-old subjects. Eur J Haematol.

[41] Duron E, Vidal JS, Funalot B, Brunel N, Viollet C, Rigaud AS (2015). Insulin-like growth factor-i, insulin-like growth factor binding protein-3 and blood hemoglobin concentration in an elderly population. J Gerontol A Biol Sci Med Sci.

[42] De Vita F, Maggio M, Lauretani F, Crucitti L, Bandinelli S, Mammarella F (2015). Insulin-like growth factor-1 and anemia in older subjects: the inchianti study. Endocr Pract.

[43] Maggio M, De Vita F, Fisichella A, Lauretani F, Ticinesi A, Ceresini G (2015). The role of the multiple hormonal dysregulation in the onset of "anemia of aging": focus on testosterone, IGF-1, and thyroid hormones. Int J Endocrinol.

[44] Schop A, Stouten K, Riedl J, van Houten R, van Rosmalen J, Wolfhagen F (2019). Long-term outcomes in patients newly diagnosed with iron deficiency anaemia in general practice: a retrospective cohort study. BMJ Open.

[45] Kan E, Kan EK, Atmaca A, Atmaca H, Colak R (2013). Visual field defects in 23 acromegalic patients. Int Ophthalmol.

[46] Civil A, van Genesen ST, Klok EJ, Lubsen NH (2000). Insulin and IGF-I affect the protein composition of the lens fibre cell with possible consequences for cataract. Exp Eye Res.

[47] Pivonello R, Auriemma RS, Grasso LF, Pivonello C, Simeoli C, Patalano R (2017). Complications of acromegaly: cardiovascular, respiratory and metabolic comorbidities. Pituitary.

[48] Tabák AG, Jokela M, Akbaraly TN, Brunner EJ, Kivimäki M, Witte DR (2009). Trajectories of glycaemia, insulin sensitivity, and insulin secretion before diagnosis of type 2 diabetes: an analysis from the Whitehall II study. Lancet.

[49] Aissani B, Zhang K, Wiener H (2015). Genetic determinants of uterine fibroid size in the multiethnic NIEHS uterine fibroid study. Int J Mol Epidemiol Genet.

[50] Gadelha MR, Kasuki L, Lim DST, Fleseriu M (2019). Systemic complications of acromegaly and the impact of the current treatment landscape: an update. Endocr Rev.

[51] Firth SM, Baxter RC (2002). Cellular actions of the insulin-like growth factor binding proteins. Endocr Rev.

